# Continuous Infusion of Piperacillin/Tazobactam in Septic Critically Ill Patients—A Multicenter Propensity Matched Analysis

**DOI:** 10.1371/journal.pone.0049845

**Published:** 2012-11-21

**Authors:** João Gonçalves-Pereira, Bruno Serra Oliveira, Sérgio Janeiro, Joana Estilita, Catarina Monteiro, Andrea Salgueiro, Alfredo Vieira, Joao Gouveia, Carolina Paulino, Luis Bento, Pedro Póvoa

**Affiliations:** 1 Unidade de Cuidados Intensivos Polivalente, Hospital São Francisco Xavier Hospital, CHLO, Lisboa, Portugal; 2 CEDOC, Faculdade de Ciências Médicas, Universidade Nova de Lisboa, Lisboa, Portugal; 3 Serviço de Medicina Intensiva, Hospital Santa Maria, CHLN, Lisboa, Portugal; 4 Unidade de Urgência Médica, Hospital São José, CHLC, Lisboa, Portugal; 5 Unidade de Cuidados Intensivos, Hospital Barlavento Algarvio, Portimão, Portugal; 6 Serviço de Medicina Intensiva, Hospital Geral, CHUC, Coimbra, Portugal; 7 Unidade de Cuidados Intensivos, Hospital do Santo Espírito, Évora, Portugal; 8 Unidade de Cuidados Intensivos, Hospital Pulido Valente, CHLN, Lisboa, Portugal; D’or Institute of Research and Education, Brazil

## Abstract

The clinical efficacy of continuous infusion of piperacillin/tazobactam in critically ill patients with microbiologically documented infections is currently unknown. We conducted a retrospective multicenter cohort study in 7 Portuguese intensive care units (ICU). We included 569 critically ill adult patients with a documented infection and treated with piperacillin/tazobactam admitted to one of the participating ICU between 2006 and 2010. We successfully matched 173 pairs of patients according to whether they received continuous or conventional intermittent dosing of piperacillin/tazobactam, using a propensity score to adjust for confounding variables. The majority of patients received 16g/day of piperacillin plus 2g/day of tazobactam. The 28-day mortality rate was 28.3% in both groups (p = 1.0). The ICU and in-hospital mortality were also similar either in those receiving continuous infusion or intermittent dosing (23.7% vs. 20.2%, p = 0.512 and 41.6% vs. 40.5%, p = 0.913, respectively). In the subgroup of patients with a Simplified Acute Physiology Score (SAPS) II>42, the 28-day mortality rate was lower in the continuous infusion group (31.4% vs. 35.2%) although not reaching significance (p = 0.66). We concluded that the clinical efficacy of piperacillin/tazobactam in this heterogeneous group of critically ill patients infected with susceptible bacteria was independent of its mode of administration, either continuous infusion or intermittent dosing.

## Introduction

The primary determinant of piperacillin/tazobactam efficacy is the amount of time in which the non-protein bound drug concentration (*f*T) exceeds the minimum inhibitory concentration (MIC) of the microorganism (*f*T>MIC) [1]. Piperacillin/tazobactam was approved to be administered by intermittent dosing. However with intermittent dosing, β-lactams attain a high peak concentration, but the presence of increased clearance can lead to a short half-life and a sub-optimal *f*T>MIC [2], [3]. Furthermore, optimizing *f*T>MIC is particularly difficult for microorganisms with elevated MICs.

Pharmacokinetic studies have shown that prolonging the infusion time provides more consistent serum levels and maximizes *f*T>MIC [4], [5]. It is unclear, however, if this approach could be translated into better patient outcomes.

Several trials comparing clinical outcomes of extended or continuous infusion of β-lactams with intermittent dosing have been completed, with conflicting results [6], [7], [8]. Moreover the interpretation of those studies remains controversial, as most trials were single centre studies, conducted with a small number of patients and failed to control for potentially confounding variables, such as focus of infection or length of hospital stay before inclusion. Besides, two recent meta-analyses failed to show any clinical benefit of extended or continuous infusion of β-lactam antibiotics in these unselected hospitalized patients [9], [10] suggesting the need to define sub-groups of patients who might benefit from this strategy.

In clinical practice continuous or extended infusion of piperacillin/tazobactam is often recommended [2], [11]. Therefore we intended to analyse if this approach may lead to a significant clinical benefit in an unselected population of critically ill patients.

We performed a multicenter propensity matched analysis comparing continuous infusion with intermittent dosing of piperacillin/tazobactam in critically ill patients with microbiologically documented infections to determine if continuous infusion resulted in improved 28-day survival, compared to conventional intermittent dosing.

## Materials and Methods

### Setting

This study was performed in 7 adult Intensive Care Units (ICU) in Portugal.

All patients admitted to one of the participant ICUs between 1^st^ January 2006 and 31^st^ December 2010 were eligible for analysis if they received piperacillin/tazobactam during their ICU stay to treat a microbiologically documented sepsis with one or more microorganisms with *in vitro* susceptibility to piperacillin/tazobactam. Microbiologically documented sepsis was considered when a relevant microorganism from a suspected focus of infection was isolated and/or bacteraemia was present. Further inclusion criteria included receiving at least 24 hrs of the studied antibiotic in the ICU.

Piperacillin/tazobactam was administered either by 30 min intermittent dosing or by continuous infusion, after an initial bolus of 4.5 g, according to the attending physician.

We excluded patients with infections caused by piperacillin/tazobactam resistant microorganisms (according to *in vitro* testing), with renal failure (defined as the need of renal replacement therapy during their ICU stay), or with incomplete clinical data. All patients were followed until death or hospital discharge.

Each patient could be included only once. Repeated admissions were discarded from further analysis and only the first admission was considered.

All data from this population were included in a database created specifically to this study.

The Institutional Review Board and Ethics Committee of all participating hospitals approved the research protocol and waived the need for written informed consent due to the observational nature of the study.

### Design

We conducted a retrospective propensity matched cohort study using prospectively collected data. We divided the patient population according to whether they received piperacillin/tazobactam by continuous infusion or conventional intermittent dosing. For the purpose of the study we considered the form of administration in the first 48 hrs.

The primary endpoint was the 28-day all cause mortality. Secondary endpoints were ICU and hospital mortality and length of stay.

### Definitions

Demographic data was collected. Site of admission was classified as emergency room, ward or another ICU. Primary source of infection was divided in lung, intra-abdominal, genito-urinary, skin and soft tissue, endovascular (including endocarditis and central venous catheter infections), central nervous system and others. The length of time in the ICU before the diagnosis of infection was divided in 4 sub-groups: less than 72 hrs, 3–7 days; 8–28 days and more than 28 days. Microbiological isolates were aggregated in Gram positive, non fermentative Gram negative or other Gram negative bacteria.

Patients were classified according to the use of systemic steroids, vasopressors and the need of mechanical ventilation.

### Matching by Propensity Score

As this was a nonrandomized study, there was a possibility that there were inherent differences between the two groups. To overcome these limitations, we used a propensity score to match patients according to the mode of piperacillin/tazobactam administration. By using propensity scores, one can better control for the likelihood of being assigned to a group and therefore reduce occult bias [12]. In our study, we modelled the likelihood of receiving continuous infusion therapy using logistic regression. We included in our regression model gender, age, severity of illness at ICU admission assessed by the Simplified Acute Physiology Score (SAPS) II score [13], admission from emergency room, ward or another ICU, the use of vasopressors, systemic steroids, invasive mechanical ventilation, source of infection, microbiological isolate and length of time in the ICU before starting piperacillin/tazobactam.

This analysis allowed the calculation of the probability of receiving piperacillin/tazobactam by continuous infusion for each patient. The propensity score area under the receiver operating characteristic curve was 0.74, indicating good discrimination (Hosmer and Lemeshow goodness of fit test, p = 0.741).

Subsequently, patients who received piperacillin/tazobactam by continuous infusion were matched with patients treated with intermittent dosing with the nearest propensity score (within a range of 0.01 on a scale from 0 to 1), using a neighbour matching methodology. Matching was performed without knowledge of the patients’ outcomes.

The success of this matching was assessed by evaluating differences in individual demographic data (Table 1).

**Table 1:** Demographic data from the selected matched cohort.

	Intermittent Dosing (n = 173)	Continuous Infusion (n = 173)	P Value
**Male Sex**	114 (65.9%)	114 (65.9%)	1.0*
**Age** (years)	60.7±18.2	60.8±18.9	0.94**
**Local of admission**			0.803*
Emergency room	106 (61.3%)	107 (61.8%)	
Ward	64 (37%)	62 (35.8%)	
Another ICU	3 (1.7%)	4 (2.3%)	
**SAPS II**	47.7±14.7	47.5±14.8	0.909**
**Time in the ICU before piperacillin/tazobactam**			0.845*
<72 hrs	96 (55.5%)	94 (54.3%)	
3–7 days	42 (24.3%)	38 (22%)	
8–28 days	30 (17.3%)	35 (20.2%)	
>28 days	5 (2.9%)	6 (3.5%)	
**Systemic steroids**	80 (46.2%)	87 (50.3%)	0.5*
**Vasopressors**	97 (56.1%)	96 (55.5%)	1.0*
**Invasive mechanical ventilation**	162 (93.6%)	159 (91.3%)	0.557*
**Infection source**			0.739*
Lung	125 (72.3%)	120 (69.4%)	
Intra-abdominal	22 (12.7%)	21 (12.1%)	
Genito-Urinary	14 (8.1%)	16 (9.2%)	
Skin and Soft tissue	4 (2.3%)	4 (2.3%)	
Blood stream infections	8 (4.6%)	12 (6.9%)	
**Isolated bacteria**			0.387*
NFGNB	79 (45.7%)	78 (45.1%)	
Other Gram negative	64 (37%)	62 (35.8%)	
Gram positive	30 (17.3%)	33 (19.1%)	

SAPS II - Simplified Acute Physiology Score; ICU – Intensive Care Unit; NFGNB – Non-fermenting Gram-negative bacteria.

Data presented as mean ± standard deviation or N (percentage).

* McNemar’s test;

** Paired Student’s *t* test.

After matching was completed, this new data set constituted our study population to assess the effect of piperacillin/tazobactam mode of administration on outcomes.

### Statistical Analysis

Descriptive statistical analysis was performed. Continuous variables were expressed as median [interquartile range] or mean ± standard deviation according to data distribution. Comparisons between infusion groups were performed with paired Student’s *t* test for continuous variables or McNemar’s test with continuity correction for categorical variables, to account for the matched design.

Cumulative mortality was calculated for continuous and intermittent dosing groups during the first 28 days after receiving the first dose of piperacillin/tazobactam.

Sub-group analyses were performed for patients with pneumonia, for patients treated with monotherapy, for those receiving vasopressors and according to the isolated microorganism (*Pseudomonas aeruginosa*, Gram positive or other Gram negative bacteria). In order to evaluate a potential mortality benefit of continuous infusion in the sub-group of patients with the higher severity scores, as described by Lodise et al [14], we performed a similar analysis using the SAPS II score. Accordingly we stratified the patients to identify the SAPS II cut-off, which allowed splitting the data and select the sub-group with the largest 28-day mortality difference between patients receiving either continuous infusion or intermittent dosing of piperacillin/tazobactam. Chi-square test was used to test association between type of infusion and mortality in these sub-groups. The Mann-Whitney *U* test was used to test association between the type of infusion and the length of stay in patients who were discharged from the ICU and the Hospital.

Data were analyzed using PASW Statistics v.18.0 (SPSS, Chicago, IL). All statistics were two-tailed, and significance level was defined as p<0.05.

## Results

During the study period a total of 569 patients admitted to one of the participating ICUs received at least 24 hrs of piperacillin/tazobactam to treat a microbiologically documented sepsis.

According to the propensity score a total of 173 pairs (N = 346 patients, 61% of total population) were successfully matched and enrolled in the study. Groups were well balanced as shown in Table 1.

The main focus of infection was the lung, which constituted 70.8% of all infections. Gram-negative bacteria were predominant (81.8%) in all studied focus of infection (Table 2). *Pseudomonas aeruginosa* was the most common isolate overall (34.4%). Besides *Staphylococcus aureus* (N = 28), *Escherichia coli* (N = 47) and *Klebsiella pneumoniae* (N = 37) were also commonly found.

**Table 2:** Main microorganisms isolated.

		Intermittent Dosing		Continuous Infusion	
**Lung**					
**Gram-negative**		**104**		**98**	
* Enterobacter cloacae*		10		4	
* Escherichia coli*		14		9	
* Haemophilus influenzae*		8		10	
* Klebsiella pneumoniae*		14		9	
* Pseudomonas aeruginosa*		45		45	
Other NFGNB		3		4	
Other Gram-negative		10		17	
**Gram-positive**		**21**		**22**	
* Staphylococcus aureus*		11		14	
* Streptococcus pneumoniae*		5		7	
Other Gram-positive		5		1	
**Abdomen**					
**Gram-negative**		**20**		**20**	
* Escherichia coli*		2		6	
* Klebsiella pneumoniae*		4		5	
* Pseudomonas aeruginosa*		12		7	
Other Gram-negative		2		2	
**Gram-Positive**		**2**		**1**	
**Other**					
**Gram-negative**	**18**		**23**		
* Escherichia coli*	4		12		
* Klebsiella pneumoniae*	3		2		
* Pseudomonas aeruginosa*	4		6		
Other Gram-negative	7		3		
**Gram-Positive**	**8**		**9**		
* Enterococcus faecalis*	2		3		
* Staphylococcus aureus*	4		5		
Other Gram-positive	2		1		

All included microorganisms had *in vitro* susceptibility to piperacillin/tazobactam.

NFGNB – Non-fermenting Gram-negative bacteria.

The vast majority of patients in both groups received 16 g of piperacillin plus 2 g of tazobactam per day (80.9% of the intermittent dosing group and 79.2% of the continuous infusion group). The mean daily doses of piperacillin were 14.9 g and 14.8 g, respectively (p = 0.84). A second antibiotic, effective against the isolated microorganism, was given to 31.2% of patients, 29.5% of those receiving intermittent dosing and 32.9% continuous infusion (p = 0.77).

**Figure 1:**
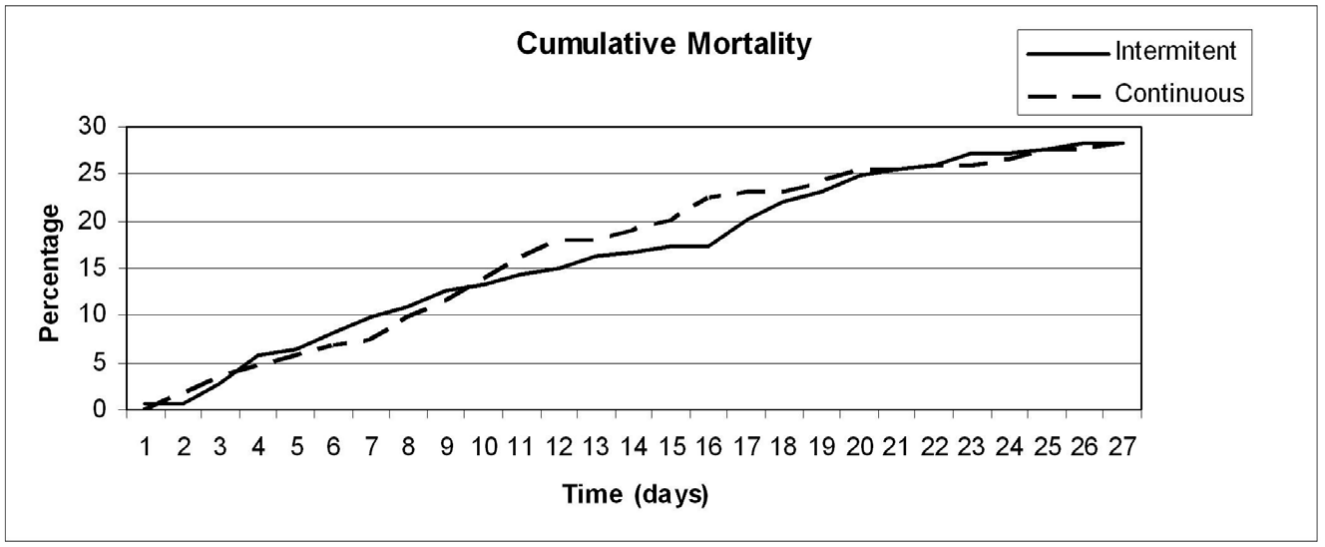
Cumulative mortality in the first 28 days after starting piperacillin/tazobactam therapy either in continuous infusion (dashed line) or 30 min bolus dosing (solid line).

A total of 98 patients (28.3%) died in the first 28 days after starting piperacillin/tazobactam. No differences were found related to the piperacillin/tazobactam mode of administration, with 49 deaths in each group (p = 1.0). Mortality in the ICU was also similar (continuous infusion 23.7% and intermittent dosing 20.2%, p = 0.512) as well as in-hospital mortality, 41.6% and 40.5% respectively (p = 0.913) – Table 3. Cumulative mortality in the first 28 days after starting piperacillin/tazobactam is shown in Figure 1. No significant differences in 28-day mortality were also noted in any of the studied sub-groups (Table 4).

**Table 3:** Outcomes of patients treated with piperacillin/tazobactam, either as continuous infusion or intermittent dosing.

	Continuous Infusion (n = 173)	Intermittent dosing (n = 173)	P value
**28-day mortality**	28.3%	28.3%	1.0*
**ICU mortality**	23.7%	20.2%	0.512*
**In-Hospital mortality**	41.6%	40.5%	0.913**
**Length of ICU stay** 1	12 [14]	11.5 [11]	0.582**
**Length of Hospital stay** 2	30 [32]	31 [54]	0.475**
**SAPS II>42 (N, 28 day mortality)**	105, 31.4%	108, 35.2%	0.66#

1 Patients discharged from ICU;

2 Patients discharged from hospital.

ICU – Intensive Care Unit; SAPS - Simplified Acute Physiology Score.

Data presented as percentage or median [Interquartile Range].

* McNemar’s test;

** Mann Whitney *U* test;

# Chi Square test.

**Table 4:** Sub-group analyses of 28-day mortality.

	N	28-day Mortality	P value
**Vasopressors**			0.88
Intermittent dosing	97	32%	
Continuous infusion	96	30.2%	
**Anbitiotic Monotherapy**			0.86
Intermittent dosing	116	28.4%	
Continuous infusion	122	27.0%	
**Pneumonia**			0.85
Intermittent dosing	125	29.6%	
Continuous infusion	120	30.3%	
***P aeruginosa*** ** Infection**			0.84
Intermittent dosing	61	29.5%	
Continuous infusion	58	32.8%	
**Gram positive bacteria**			1.0
Intermittent dosing	30	30.0%	
Continuous infusion	33	30.3%	
**Other Gram negative** **bacteria**			0.86
Intermittent dosing	82	26.8%	
Continuous infusion	82	24.4%	

Stratifying patients according to SAPS II score allowed us to identify the sub-group of patients with the largest 28-day mortality difference. Patients with a SAPS II score above 42, had a 28-day mortality rate of 35.2% when receiving piperacillin/tazobactam by intermittent dosing and 31.4% if receiving continuous infusion, but again this difference was not statistically significant (p = 0.66).

Among the patients discharged from the ICU, the median length of ICU stay was similar for both groups (12.0 and 11.5 days, respectively). In-hospital duration of stay was also not different (Table 3).

## Discussion

In this study we addressed the use of piperacillin/tazobactam for the treatment of microbiologically documented infections caused by susceptible species in critically ill patients. In this cohort of patients, mostly treated with 16g/day of piperacillin plus 2g/day of tazobactam, we found continuous infusion to be as effective as conventional intermittent dosing, even in the most severe patient sub-group.

The β-lactam antibiotics, including piperacillin/tazobactam, due to their large antimicrobial spectrum and low toxicity, are among the first line therapy in critically ill septic patients. There is large evidence that its pharmacodynamic target, associated with the maximal microbiological effect, is the time during which the non-protein bound drug concentration exceeds the MIC of the organism (*f*T>MIC) [11]. Therefore extended or even continuous infusion of β-lactam antibiotics has gained enthusiasm, since an improved profile of β-lactams and a longer bacterial exposure can be expected [15], [16]. Several studies reported small benefits of continuous infusion which has further been supported by pharmacodynamic modelling studies [9].

Continuous infusion has also been shown to increase piperacillin/tazobactam lung epithelial lining fluid concentration [17] but only in patients with normal renal function. No differences were found in patients with moderate renal failure (creatinine clearance<50 mL/min) and no survival benefit has been reported [17].

In a retrospective cohort study of patients with ventilator-associated pneumonia, Lorente et al [18] identified a higher survival rate in patients receiving continuous infusion (90.5%) compared with conventional intermittent dosing, 59.6%. Reduced mortality with extended infusion piperacillin/tazobactam (4-hour infusion) was also described in a single-center cohort study of 194 seriously ill patients with *Pseudomonas aeruginosa* infection [14]. However, in this study, only in the subset of the more severe patients (according to an Acute Physiology and Chronic Health Evaluation II score [19] higher than 17) a lower 14-day mortality rate was associated with extended infusion (12.2% vs. 31.6%; p = 0.04).

In contrast to our study, both these studies were single centre, addressing only a specific population, ventilator associated pneumonia [18] and *Pseudomonas aeruginosa* infection [14]. Moreover patients were not matched according to clinical variables and unknown bias may have been introduced.

Pharmacodynamic modelling had also suggested a potential benefit of extended or continuous infusion of β-lactams antibiotics in outcome, but this was especially noted for bacteria with a high MIC, near the antibiotic susceptibility breakpoint [20], [21]. Although we did not evaluate the MICs of all isolated bacteria, we exclude patients with resistant or intermediate isolated microorganisms.

A recently published randomized control single center trial compared another β-lactam, meropenem, either in continuous infusion or in intermittent dosing, in 240 ICU young patients (mean age 46 years), mostly with hospital acquired infections [7]. There was no survival benefit in the overall population (83.0% in the continuous infusion group and 75.0% in the intermittent dosing group; p = 0.18) or in the sub-group with the higher APACHE II score (75.5% vs. 79.2%; p = 0.81). Although patients in the intermittent dosing group received a high meropenem dose (6g/day), those who receive continuous infusion had a higher rate of microbiological success [Odds Ratio - 2.98 (95% confidence interval 1.05 to 8.44; p = 0.04] and a lower length of stay in the ICU, despite receiving a lower total dose of meropenem.

Two recently published meta-analysis of randomized, prospective studies also failed to show any survival benefit of the mode of β-lactam antibiotics administration, extended or continuous infusion [9], [10] with odds ratio of 1.0 and 0.92, respectively.

To identify any subset of patients who had a significant mortality benefit of continuous infusion in our study, we stratify our population according to their severity scores, as was described in the study of Lodise et al [14]. Accordingly we found that the largest mortality difference was in the subset of patients with a SAPS II>42. Using that cut-off, a non significant lower mortality rate (31.4% vs. 35.2%) in patients receiving continuous infusion was noted. To adequately confirm this difference (with a power of 80%), a study including 4822 patients would have been needed.

The clinical benefits of continuous infusion of piperacillin/tazobactam may be mostly noted in patients infected with bacteria with high MIC, in whom conventional intermittent dosing may fail to achieve the pharmacodynamic target (*f*T>MIC), in immunocompromissed patients [22] and in patients with increased β-lactam clearance (closely related to creatinine clearance), who may also be at risk of underdosing and, consequently, therapeutic failure [23], [24], [25]. However the use of high β-lactam antibiotics dose (as in the study of Chytra et al [7]) may overcome any potential limitations of intermittent dosing. This could have also contributed to our findings.

An increase in creatinine clearance, which is usually related to a shorter *f*T>MIC of β-lactam antibiotics, has been shown to be a common finding in septic surgical or trauma patients [26] and also in medical patients [27]. An effort to identify easily available clinical markers of patients at risk for underdosing, who might benefit from an improved pharmacokinetic dosing guidance (including continuous infusion of β-lactam antibiotics) is currently underway [28], [29].

A large volume of distribution is also common in critically ill septic patients. Therefore failure to give an appropriate loading dose may also lead to an initially low *f*T>MIC [30]. However, due to the fact that volume of distribution and half-life are directly proportional, the enhanced volume of distribution can increase drug half-life (and consequently *f*T>MIC) [31], as long as drug clearance remains unchanged.

Critically ill patients with organ dysfunction commonly experience drug accumulation and toxicity [32] and this may easily go unrecognized [33], [34]. Consequently therapeutic drug monitoring has been proposed as a valuable tool to help guide antibiotic therapy, unveiling both under and overdosing [35], [36]. Presently this is only commonly available for aminoglycosides and vancomycin. Furthermore, better guidance of the ideal therapeutic targets of β-lactam antibiotics are probably needed [37].

As far as we are aware, this is the largest study evaluating the outcomes of patients treated with continuous infusion of a β-lactam antibiotic, evaluating 346 patients. Moreover it is a multicenter study which included critically ill patients with different microbiologically documented infections caused by bacteria susceptible to the study drug, matched by a propensity based analysis. We believe that this data suggests that the use of continuous infusion of 16g/day of piperacillin plus 2g/day of tazobactam in a heterogeneous group of critically ill patients is not associated with a decrease in mortality. Studies to identify sub-groups of critically ill patients who could benefit from this strategy are warranted.

However our study has also some limitations. First we did not measure piperacillin/tazobactam concentrations. Also, data on bacteria MIC was not available for analysis. Therefore we were not able to identify patients who did not attain the piperacillin/tazobactam pharmacodynamic target or who had toxic concentrations. We also cannot exclude an eventual difference of efficacy of continuous infusion of piperacillin/tazobactam in patients infected with borderline resistant bacteria, as we only included patients with susceptible bacteria. Second this was a retrospective study, addressing a heterogeneous ICU population and, despite the matching, some unknown bias may have occurred. Besides, we recognize that our sample may have been underpowered to unveil differences in some sub-groups of interest. Finally we did not analyse data on antibiotic duration or recurrent infection, as several patients were discharged from the ICU while still receiving the antibiotic.

## Conclusions

In this cohort of heterogeneous critically ill patients with infections caused by susceptible bacteria, the clinical efficacy of 16g/day of piperacillin plus 2g/day of tazobactam was independent of the mode of administration, either continuous infusion or conventional intermittent dosing.

## References

[1] Drusano GL (2004) Antimicrobial pharmacodynamics: critical interactions of ‘bug and drug’. Nat Rev Microbiol 2: 289–300. 15031728 10.1038/nrmicro862

[2] Roberts J, Lipman J, Blot S, Rello J (2008) Better outcomes through continuous infusion of time-dependent antibiotics to critically ill patients? Curr Opin Crit Care 14: 390. 18614901 10.1097/MCC.0b013e3283021b3a

[3] Lipman J, Wallis SC, Rickard C (1999) Low plasma cefepime levels in critically ill septic patients: pharmacokinetic modeling indicates improved troughs with revised dosing. Antimicrob Agents Chemother 43: 2559–2561. 10508045 10.1128/aac.43.10.2559 PMC89521

[4] De Jongh R, Hens R, Basma V, Mouton JW, Tulkens PM, et al (2008) Continuous versus intermittent infusion of temocillin, a directed spectrum penicillin for intensive care patients with nosocomial pneumonia: stability, compatibility, population pharmacokinetic studies and breakpoint selection. J Antimicrob Chemother 61: 382–388. 18070831 10.1093/jac/dkm467

[5] Roberts JA, Kirkpatrick CM, Roberts MS, Dalley AJ, Lipman J (2009) First-dose and steady-state population pharmacokinetics and pharmacodynamics of piperacillin by continuous or intermittent dosing in critically ill patients with sepsis. Int J Antimicrob Agents 35: 156–163. 20018492 10.1016/j.ijantimicag.2009.10.008

[6] Georges B, Conil JM, Seguin T, Dieye E, Cougot P, et al (2008) Cefepime in intensive care unit patients: validation of a population pharmacokinetic approach and influence of covariables. Int J Clin Pharmacol Ther 46: 157–164. 18397688 10.5414/cpp46157

[7] Chytra I, Stepan M, Benes J, Pelnar P, Zidkova A, et al (2012) Clinical and microbiological efficacy of continuous versus intermittent application of meropenem in critically ill patients: a randomized open-label controlled trial. Crit Care 16: R113. 22742765 10.1186/cc11405 PMC3580671

[8] Roberts JA, Boots R, Rickard CM, Thomas P, Quinn J, et al (2007) Is continuous infusion ceftriaxone better than once-a-day dosing in intensive care? A randomized controlled pilot study. J Antimicrob Chemother 59: 285–291. 17135183 10.1093/jac/dkl478

[9] Roberts JA, Webb S, Paterson D, Ho KM, Lipman J (2009) A systematic review on clinical benefits of continuous administration of beta-lactam antibiotics. Crit Care Med 37: 2071–2078. 19384201 10.1097/CCM.0b013e3181a0054d

[10] Tamma PD, Putcha N, Suh YD, Van Arendonk KJ, Rinke ML (2011) Does prolonged beta-lactam infusions improve clinical outcomes compared to intermittent infusions? A meta-analysis and systematic review of randomized, controlled trials. BMC Infect Dis 11: 181. 21696619 10.1186/1471-2334-11-181 PMC3141415

[11] Roberts JA, Lipman J (2009) Pharmacokinetic issues for antibiotics in the critically ill patient. Crit Care Med 37: 840–851; quiz 859. 10.1097/CCM.0b013e3181961bff 19237886

[12] Joffe MM, Rosenbaum PR (1999) Invited commentary: propensity scores. Am J Epidemiol 150: 327–333. 10453808 10.1093/oxfordjournals.aje.a010011

[13] Le GallJR, Lemeshow S, Saulnier F (1993) A new Simplified Acute Physiology Score (SAPS II) based on a European/North American multicenter study. JAMA 270: 2957–2963. 8254858 10.1001/jama.270.24.2957

[14] Lodise TP Jr, Lomaestro B, Drusano GL (2007) Piperacillin-tazobactam for Pseudomonas aeruginosa infection: clinical implications of an extended-infusion dosing strategy. Clin Infect Dis 44: 357–363. 17205441 10.1086/510590

[15] Nicolau DP (2008) Pharmacodynamic optimization of beta-lactams in the patient care setting. Crit Care 12 Suppl 4 S2. 10.1186/cc6818 PMC2391264 18495059

[16] Kasiakou SK, Sermaides GJ, Michalopoulos A, Soteriades ES, Falagas ME (2005) Continuous versus intermittent intravenous administration of antibiotics: a meta-analysis of randomised controlled trials. Lancet Infect Dis 5: 581–589. 16122681 10.1016/S1473-3099(05)70218-8

[17] Boselli E, Breilh D, Rimmele T, Guillaume C, Xuereb F, et al (2008) Alveolar concentrations of piperacillin/tazobactam administered in continuous infusion to patients with ventilator-associated pneumonia. Crit Care Med 36: 1500–1506. 18434883 10.1097/CCM.0b013e318170ba21

[18] Lorente L, Jimenez A, Martin MM, Iribarren JL, Jimenez JJ, et al (2009) Clinical cure of ventilator-associated pneumonia treated with piperacillin/tazobactam administered by continuous or intermittent infusion. Int J Antimicrob Agents 33: 464–468. 19150225 10.1016/j.ijantimicag.2008.10.025

[19] Knaus WA, Draper EA, Wagner DP, Zimmerman JE (1985) APACHE II: a severity of disease classification system. Crit Care Med 13: 818–829. 3928249

[20] Rafati MR, Rouini MR, Mojtahedzadeh M, Najafi A, Tavakoli H, et al (2006) Clinical efficacy of continuous infusion of piperacillin compared with intermittent dosing in septic critically ill patients. Int J Antimicrob Agents 28: 122–127. 16815689 10.1016/j.ijantimicag.2006.02.020

[21] Lipman J, Wallis SC, Rickard CM, Fraenkel D (2001) Low cefpirome levels during twice daily dosing in critically ill septic patients: pharmacokinetic modelling calls for more frequent dosing. Intensive Care Med 27: 363–370. 11396280 10.1007/s001340000741

[22] Bodey GP, Ketchel SJ, Rodriguez V (1979) A randomized study of carbenicillin plus cefamandole or tobramycin in the treatment of febrile episodes in cancer patients. Am J Med 67: 608–616. 495630 10.1016/0002-9343(79)90242-0

[23] Lipman J, Wallis SC, Boots RJ (2003) Cefepime versus cefpirome: the importance of creatinine clearance. Anesth Analg 97: 1149–1154. 14500173 10.1213/01.ANE.0000077077.54084.B0

[24] Shikuma LR, Ackerman BH, Weaver RH, Solem LD, Strate RG, et al (1990) Effects of treatment and the metabolic response to injury on drug clearance: a prospective study with piperacillin. Crit Care Med 18: 37–41. 2293966 10.1097/00003246-199001000-00010

[25] Conil JM, Georges B, Mimoz O, Dieye E, Ruiz S, et al (2006) Influence of renal function on trough serum concentrations of piperacillin in intensive care unit patients. Intensive Care Med 32: 2063–2066. 17061021 10.1007/s00134-006-0421-1

[26] Fuster-Lluch O, Geronimo-Pardo M, Peyro-Garcia R, Lizan-Garcia M (2008) Glomerular hyperfiltration and albuminuria in critically ill patients. Anaesth Intensive Care 36: 674–680. 18853585 10.1177/0310057X0803600507

[27] Baptista JP, Udy AA, Sousa E, Pimentel J, Wang L, et al (2011) A comparison of estimates of glomerular filtration in critically ill patients with augmented renal clearance. Crit Care 15: R139. 21651804 10.1186/cc10262 PMC3219011

[28] Roberts JA, Roberts MS, Semark A, Udy AA, Kirkpatrick CM, et al (2011) Antibiotic dosing in the ‘at risk’ critically ill patient: Linking pathophysiology with pharmacokinetics/pharmacodynamics in sepsis and trauma patients. BMC Anesthesiol 11: 3. 21333028 10.1186/1471-2253-11-3 PMC3050838

[29] Roberts JA, De Waele JJ, Dimopoulos G, Koulenti D, Martin C, et al (2012) DALI: Defining Antibiotic Levels in Intensive care unit patients: a multi-centre point of prevalence study to determine whether contemporary antibiotic dosing for critically ill patients is therapeutic. BMC Infect Dis 12: 152. 22768873 10.1186/1471-2334-12-152 PMC3506523

[30] Taccone FS, Laterre PF, Dugernier T, Spapen H, Delattre I, et al (2010) Insufficient beta-lactam concentrations in the early phase of severe sepsis and septic shock. Crit Care 14: R126. 20594297 10.1186/cc9091 PMC2945087

[31] Bourget P, Lesne-Hulin A, Le Reveille R, Le Bever H, Carsin H (1996) Clinical pharmacokinetics of piperacillin-tazobactam combination in patients with major burns and signs of infection. Antimicrob Agents Chemother 40: 139–145. 8787895 10.1128/aac.40.1.139 PMC163072

[32] Ulldemolins M, Roberts JA, Lipman J, Rello J (2011) Antibiotic dosing in multiple organ dysfunction syndrome. Chest 139: 1210–1220. 21540219 10.1378/chest.10-2371

[33] Chapuis TM, Giannoni E, Majcherczyk PA, Chiolero R, Schaller MD, et al (2010) Prospective monitoring of cefepime in intensive care unit adult patients. Crit Care 14: R51. 20359352 10.1186/cc8941 PMC2887166

[34] Singer M, Glynne P (2005) Treating critical illness: the importance of first doing no harm. PLoS Med 2: e167. 15971943 10.1371/journal.pmed.0020167 PMC1160576

[35] Roberts JA, Ulldemolins M, Roberts MS, McWhinney B, Ungerer J, et al (2010) Therapeutic drug monitoring of beta-lactams in critically ill patients: proof of concept. Int J Antimicrob Agents 36: 332–339. 20685085 10.1016/j.ijantimicag.2010.06.008

[36] Sime FB, Roberts MS, Peake SL, Lipman J, Roberts JA (2012) Does Beta-lactam Pharmacokinetic Variability in Critically Ill Patients Justify Therapeutic Drug Monitoring? A Systematic Review. Annals of Intensive Care 2: 35. 22839761 10.1186/2110-5820-2-35 PMC3460787

[37] Goncalves-Pereira J, Povoa P (2011) Antibiotics in critically ill patients: a systematic review of the pharmacokinetics of beta-lactams. Crit Care 15: R206. 21914174 10.1186/cc10441 PMC3334750

